# Case Report: SMARCA4-deficient non-small cell lung cancer — effective local control with radiotherapy and emergence of a targetable EGFR mutation

**DOI:** 10.3389/fonc.2026.1724204

**Published:** 2026-03-25

**Authors:** Jingru Luo, Shu Lin, Mingfa Wang, Yuecan Zeng, Wenjun Tang

**Affiliations:** 1 Department of Oncology, The Second Affiliated Hospital of Hainan Medical University, Haikou, Hainan, China; 2 Department of Pathology, The Second Affiliated Hospital of Hainan Medical University, Haikou, Hainan, China; 3 Department of Radiation Oncology, The Second Affiliated Hospital of Hainan Medical University, Haikou, Hainan, China

**Keywords:** EGFR L858R/T790M, immunotherapy, NSCLC, radiotherapy, SMARCA4 deficiency

## Abstract

**Background:**

SMARCA4-deficient non-small cell lung cancer (SMARCA4-dNSCLC) represents a distinct, highly aggressive molecular subtype associated with a poor prognosis. While immune checkpoint inhibitors offer potential benefits, therapeutic options remain limited. Notably, SMARCA4 alterations are typically mutually exclusive with classic driver mutations (e.g., EGFR, ALK), rendering targeted therapies generally inapplicable at diagnosis. Consequently, the emergence of actionable driver mutations following systemic therapy is an exceptionally rare but clinically significant phenomenon in this entity.

**Case presentation:**

We present the case of a 52-year-old male diagnosed with stage T4N3M1a (IVA) SMARCA4-dNSCLC in April 2023, initially presenting with superior vena cava syndrome (SVCS). The patient was managed with a multimodal treatment strategy. Initial thoracic radiotherapy (50 Gy in 25 fractions) successfully alleviated the SVCS, enabling subsequent systemic therapy with tislelizumab, nab-paclitaxel, and carboplatin. Upon first disease progression nine months later (April 2024), he received a second course of radiotherapy (30 Gy in 10 fractions) combined with chemoimmunotherapy, achieving stable disease for five months. Following a second progression in October 2024, which was complicated by recurrent SVCS and malignant effusions, an SVC stent was placed. Critically, dynamic genetic profiling of the pleural effusion in November 2024 identified an emergent EGFR L858R mutation—a finding contrary to the typical mutual exclusivity described in this subgroup and suggests therapy-induced clonal selection. Treatment with a third-generation EGFR-TKI (aumolertinib) was initiated, resulting in significant symptomatic improvement and a progression-free survival of four months. The patient ultimately succumbed to the disease in April 2025, achieving an overall survival (OS) of 24 months. To our knowledge, this is the first report demonstrating the successful sequential integration of radiotherapy and EGFR-TKI therapy in SMARCA4-dNSCLC.

**Conclusion:**

Our experience demonstrates that a proactive, individualized multimodal strategy—integrating radiotherapy for local control and targeted therapy guided by dynamic molecular profiling—can significantly extend survival beyond the historical median of approximately 12 months for SMARCA4-deficient NSCLC.

## Introduction

Non-small cell lung cancer (NSCLC) harboring *SMARCA4* deficiency represents a distinct and aggressive molecular subtype, accounting for approximately 5% to 10% of all NSCLC cases (1–3). Although the 2021 WHO classification introduced “thoracic *SMARCA4*-deficient undifferentiated tumor” (*SMARCA4*-UT) as a separate entity, it is crucial to distinguish it from *SMARCA4*-dNSCLC *(*4). Pathologically, *SMARCA4*-dNSCLC is characterized by the retention of epithelial architecture, cellular cohesion, and diffuse strong keratin expression, features that are typically lost in *SMARCA4*-UT. Clinically, while *SMARCA4*-UT typically presents as a large mediastinal mass in younger adults, *SMARCA4*-dNSCLC predominantly affects older males with a history of smoking (5). Clinically, *SMARCA4*-dNSCLC is associated with a high tumor mutation burden (TMB) and aggressive biological behavior, including vascular invasion and early metastasis (6).

The prognosis for patients with *SMARCA4*-dNSCLC is generally poor, with studies reporting a median overall survival (OS) of approximately 12 months (7), which is significantly inferior to that of *SMARCA4*-wild-type NSCLC. Therapeutic management remains a challenge; these tumors often demonstrate resistance to standard platinum-based chemotherapy regimes (8). While immune checkpoint inhibitors (ICIs) have shown potential due to high TMB (9), outcomes are heterogeneous, and efficacy may be compromised by co-occurring mutations in genes such as *STK11* or *KEAP1*. Furthermore, *SMARCA4* mutations are typically mutually exclusive with driver mutations such as *EGFR, ALK*, and *ROS1*, limiting the applicability of targeted therapies for this subgroup (3, 10).

We present a rare case of *SMARCA4*-dNSCLC treated with multimodal therapy, including radiotherapy, immunotherapy, and chemotherapy. A pivotal finding in this case was the identification of an EGFR L858R mutation via next-generation sequencing (NGS) during disease progression—a phenomenon that challenges the conventional paradigm of mutual exclusivity between *SMARCA4* alterations and driver mutations. This finding created a unique opportunity for targeted intervention with a third-generation EGFR tyrosine kinase inhibitor (TKI). Additionally, this case demonstrates that radiotherapy can serve as a potent salvage modality for oncologic emergencies, such as tumor-related complications, in *SMARCA4*-dNSCLC. Furthermore, the emergence of an EGFR sensitizing mutation following multiline therapy suggests that targeted agents may hold clinical value in this tumor type. To our knowledge, this case illustrates a novel clinical scenario involving the application of radiotherapy and subsequent targeted therapy in a patient with *SMARCA4*-dNSCLC, providing new insights into the management of this aggressive malignancy (6).

## Case presentation

**Figure 1:**
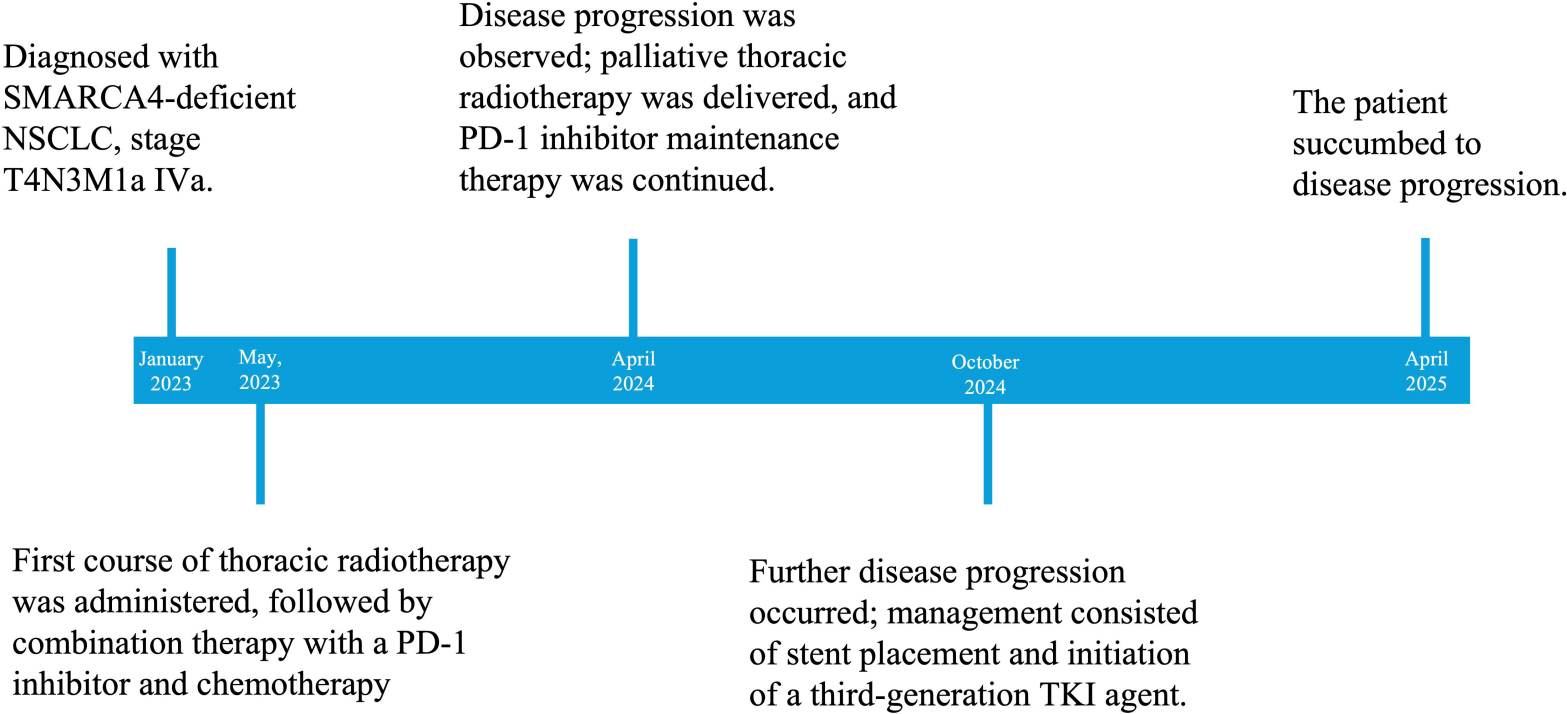
Timeline of the patient's case.

A 52-year-old male was admitted with a 4-month history of exertional chest tightness and dyspnea that resolved at rest. He denied cough, sputum, dizziness, headache, chest pain, palpitations, fever, chills, nausea, vomiting, or night sweats. Contrast-enhanced chest CT demonstrated a right hilar mass accompanied by post-obstructive pneumonia, raising suspicion for central lung cancer. Tumor thrombus was suspected within the right brachiocephalic and azygos veins. The clinical timeline is summarized in **Figure 1**.

Additional findings included emphysema with a bulla in the left upper lobe, as well as heterogeneously enhancing small mediastinal lymph nodes. Physical examination revealed facial edema and prominent superficial venous collateral vessels over the chest. No family history of malignancy was reported.

**Figure 2:**
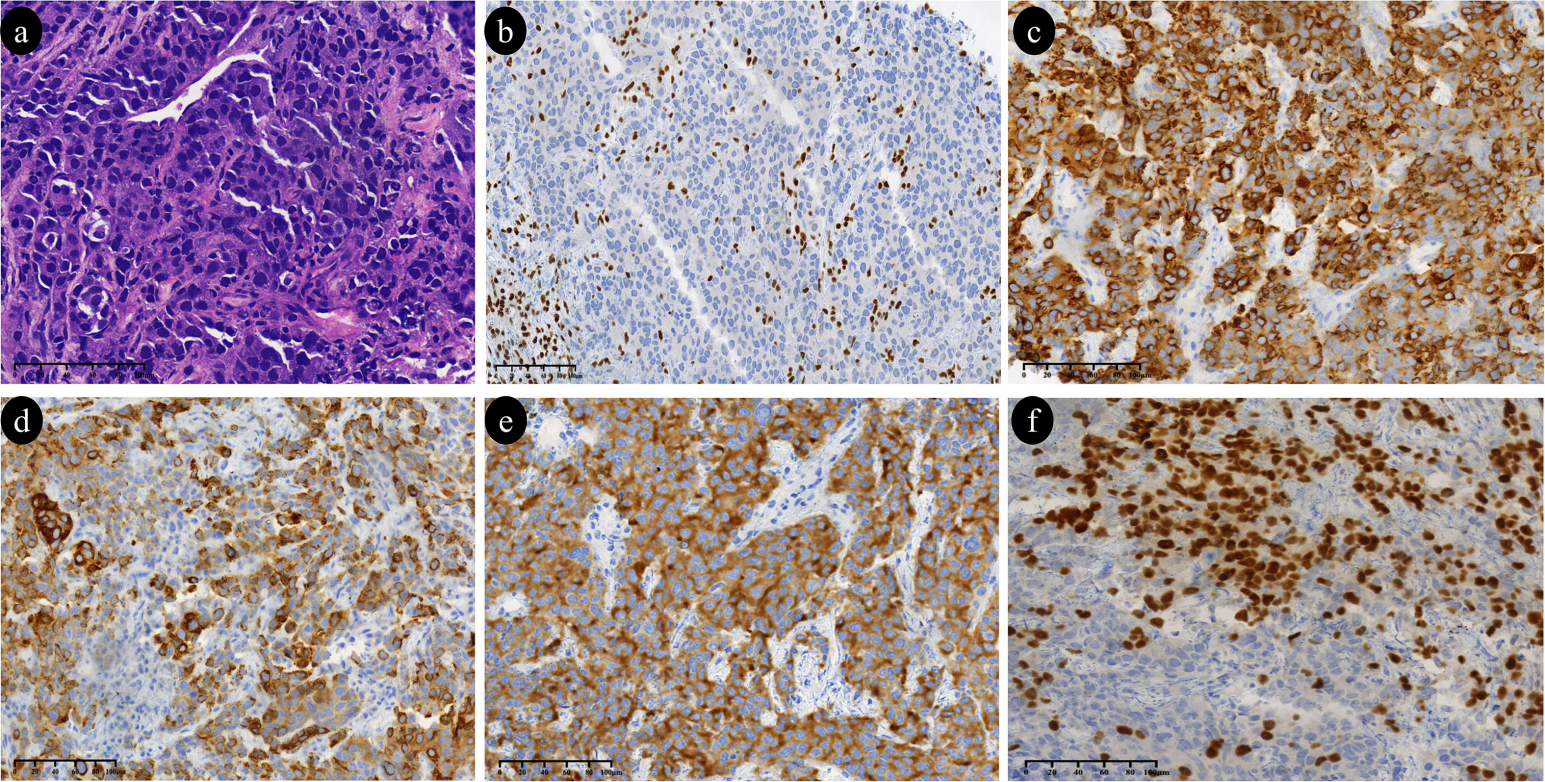
Histopathological examinations. **(a)** H&E staining: The tumor cells of the SMARCA4-deficient non-small cell lung carcinoma grow in solid sheets and exhibit marked nuclear pleomorphism and hyperchromasia (medium magnification). **(b)** SMARCA4: Loss of SMARCA4 expression in the tumor cells (MaxVision method, medium magnification). **(c)** CK (Cytokeratin): The tumor cells show positive expression for Cytokeratin (MaxVision method, medium magnification). **(d)** CK7 (Cytokeratin 7): The tumor cells show positive expression for Cytokeratin 7 (MaxVision method, medium magnification). **(e)** Syn (Synaptophysin): The tumor cells show positive expression for Synaptophysin (MaxVision method, medium magnification). **(f)** TTF-1 (Thyroid Transcription Factor-1): The tumor cells show positive expression for TTF-1 (MaxVision method, medium magnification).

A CT-guided lung biopsy was performed, revealing histologic features of sheets and nests of tumor cells exhibiting high nuclear-to-cytoplasmic ratios, enlarged and irregular nuclei, inconspicuous nucleoli, and frequent mitotic activity. Immunohistochemistry was positive for CK, CK7, and synaptophysin (Syn), with a Ki-67 index >80%. TTF-1 was partially positive. These initial findings, particularly the partial TTF-1 and CK7 positivity, were consistent with a poorly differentiated non-small cell lung carcinoma (NSCLC), although the expression of Synaptophysin (Syn) raised the differential diagnosis of large cell neuroendocrine carcinoma (LCNEC). To further characterize the tumor lineage and establish a definitive diagnosis, an expanded immunohistochemical panel was performed. Staining was negative for Vimentin, NapsinA, CK5/6, P40, P63, chromogranin A (CgA), and CD56. Crucially, the tumor cells showed a loss of *SMARCA4* (BRG1) expression. In contrast,INI-1(SMARCB1).expression was retained, albeit exhibiting a weak staining intensity. Targeted next-generation sequencing (NGS)was performed using a hybrid capture-based targeted sequencing assay on the Illumina NextSeq 2000 platform (Dingjing Biotechnology, Hangzhou, China). The assay did not identify detectable mutations in actionable drivers (*EGFR*, *ALK, ROS1)* or common co-occurring genes (such as *TP53* and *KRAS*). A diagnosis of (*SMARCA4*-dNSCLC was confirmed, staged as T4N3M1a (IVA) (**Figure 2**).

**Figure 3:**
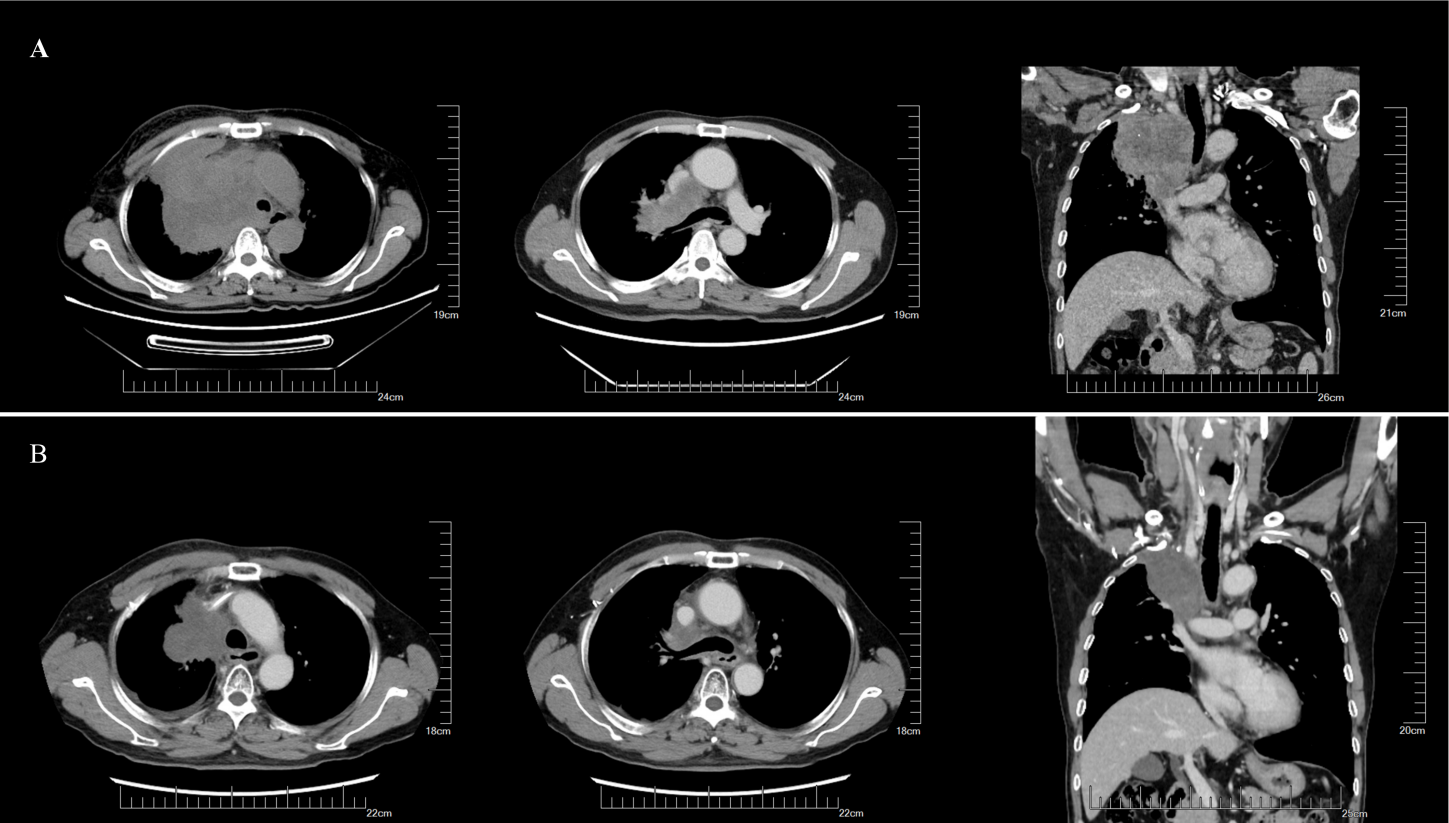
Efficacy of initial radiotherapy. **(A)** On initial presentation, enhanced CT revealed a mass in the right upper hilar region and right upper lobe with surrounding obstructive pneumonia, as well as tumor thrombus formation in the right brachiocephalic vein and azygos vein. **(B)** After radiotherapy, follow-up imaging showed significant reduction in the hilar mass.

On May 23, 2023, the patient received thoracic radiotherapy targeting the hilar mass and mediastinal lymph nodes (50 Gy in 25 fractions) with sodium glycididazole as a radiosensitizer. Radiotherapy resulted in symptomatic improvement in chest tightness, dyspnea, and facial edema. Evaluation of the follow-up imaging showed tumor regression, which was classified as stable disease (SD) according to RECIST 1.1 criteria (**Figure 3**). Although PD-L1 expression and tumor mutational burden (TMB) were not assessed due to financial constraints, immunotherapy was incorporated into the treatment regimen. This decision was based on the lack of actionable driver mutations at diagnosis, the aggressive nature of the disease, and emerging evidence suggesting potential efficacy of immune checkpoint inhibitors in this entity. He subsequently received six cycles of the anti-PD-1 antibody tislelizumab (200 mg), nab-paclitaxel (260 mg/m²), and carboplatin (AUC 5), followed by maintenance tislelizumab.

**Figure 4:**
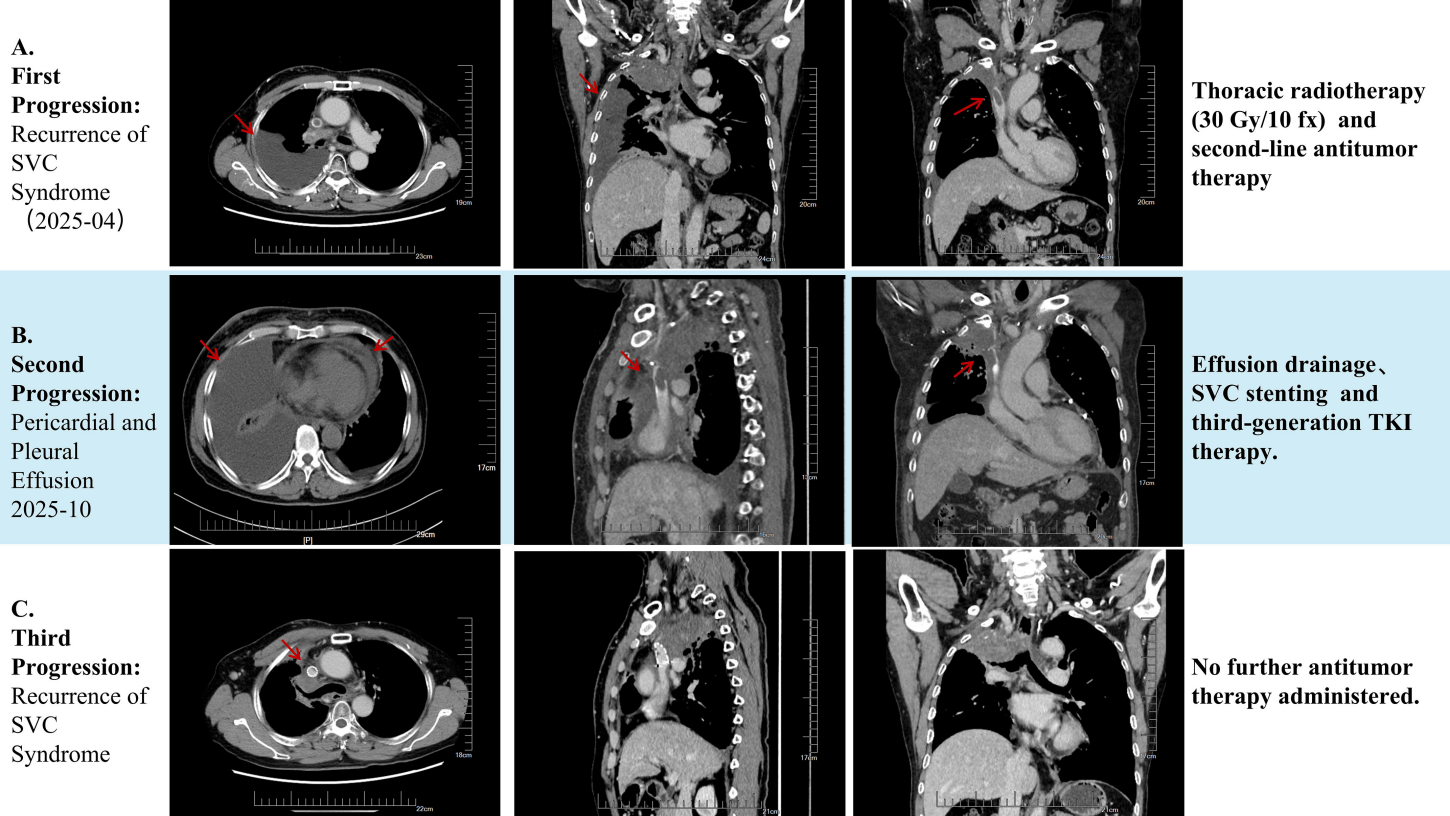
Treatment strategies administered at each disease progression. **(A)** First Progression: The patient developed superior vena cava syndrome and new-onset pleural effusion. Management consisted of a second course of thoracic radiotherapy (30 Gy in 10 fractions) combined with second-line antitumor therapy using gemcitabine and tislelizumab. The post-treatment response assessment indicated stable disease. **(B)** Second Progression: The patient experienced increased pleural effusion and new-onset pericardial effusion, accompanied by worsening chest tightness and shortness of breath. Treatment involved drainage of the serous cavity effusions, placement of a superior vena cava stent, and initiation of a third-generation tyrosine kinase inhibitor (TKI). **(C)** Third Progression: The patient had a recurrence of a tumor thrombus in the superior vena cava, with a return of chest tightness and shortness of breath. However, no further antitumor therapy was administered subsequently.

Nine months after the initiation of first-line therapy, the patient experienced a recurrence of facial and upper limb edema. Computed tomography (CT) confirmed the presence of a tumor thrombus in the superior vena cava (SVC) (**Figure 4A**). On April 24, 2024, he underwent a second course of thoracic radiotherapy (30 Gy in 10 fractions) combined with second-line systemic therapy consisting of gemcitabine and tislelizumab. Post-treatment assessment indicated stable disease. From June to September 2024, he was maintained on tislelizumab monotherapy.

In October 2024, his condition was complicated by the recurrence of SVC syndrome, accompanied by pericardial and pleural effusions(**Figure 4B**). An SVC stent was placed to relieve the obstruction, and a pleural effusion sample was sent for cytological examination and molecular profiling via NGS. While awaiting the test results, one cycle of docetaxel(75mg/m^2^) was administered. In November 2024, molecular profiling was performed on DNA extracted from the pleural effusion tumor cell block using a 23-gene lung cancer-targeted NGS panel (KingMed Diagnostics, Guangzhou, China). This analysis identified an *EGFR L858R* mutation with a variant allele frequency (VAF) of 1.38%, along with a concurrent *TP53* mutation; notably, *KRAS* remained wild-type. Subsequently, treatment with Aumolertinib (110 mg/d) was initiated. This led to significant symptomatic improvement and stable disease(SD). Unfortunately, the disease progressed after four months of therapy (**Figure 4C**), and the patient subsequently succumbed to the disease.

## Discussion

*SMARCA4*-dNSCLC is an aggressive thoracic malignancy with a propensity for widespread metastasis. Cohort studies report a median OS of approximately 12 months, with a significant proportion of patients presenting with advanced disease (11, 12). This case was diagnosed at stage IVa (T4N3M1a) and recurred as SVC tumor thrombus nine months after first-line therapy. While *SMARCA4* -dNSCLC is historically associated with poor clinical outcomes and limited response to standard therapies, the overall survival of 24 months achieved in this patient is remarkable. This significant extension of survival, far exceeding the typical median of 12 months, highlights the potential efficacy of the comprehensive multimodality therapeutic strategy employed.

Pathologically, while *SMARCA4*-UT is defined by an undifferentiated or rhabdoid phenotype (12), *SMARCA4*-dNSCLC typically manifests as adenocarcinoma or, less frequently, squamous cell carcinoma (6). However, high-grade features such as solid sheets of tumor cells with significant nuclear atypia can be present. The lung biopsy in this case showed diffuse sheets and nests of tumor cells with high nuclear-to-cytoplasmic ratios and readily identifiable mitotic figures. Immunohistochemistry confirmed loss of *SMARCA4* (BRG1) expression—a diagnostic cornerstone. Literature indicates that unlike *SMARCA4*-UT, which is typically characterized by the overexpression of stem cell markers such as SOX2 and the loss of Claudin-4, *SMARCA4*-dNSCLC often differs in its immunoprofile (13). Although these specific differential markers were not evaluated in our case, the clinical and pathological presentation aligns with the features of *SMARCA4*-dNSCLC.

Pathologically, the absence of specific squamous differentiation markers (CK5/6^−^, P40^−^, P63^−^) effectively excluded a diagnosis of squamous cell carcinoma. Concurrently, the lack of definitive glandular markers (e.g., Napsin A^−^), combined with only focal CK7 positivity and weak/partial TTF-1 expression, reflects the poorly differentiated phenotype characteristic of *SMARCA4*-dNSCLC. Notably, literature indicates that approximately 80% of TTF-1-negative or low-expressing lung adenocarcinomas harbor *SMARCA4* or SMARCA2 deletions (14). The high Ki-67 index (>80%) observed in this case is further consistent with the aggressive biological behavior of this entity.

Molecularly, *SMARCA4*-dNSCLC typically harbors a smoking-associated mutational signature (13). Large-scale genomic analyses (1) indicate a high frequency of *TP53* mutations (approx. 56%), along with common co-alterations in *KEAP1* (41%), *STK11* (39%), and *KRAS* (36%). Notably, *SMARCA4* mutations are generally mutually exclusive with typical driver mutations such as *EGFR, ALK, MET*, and *ROS1.* The emergence of an *EGFR L858R* mutation in the terminal-stage pleural effusion in this case is therefore highly unusual and may reflect clonal evolution or therapy-induced heterogeneity in the setting of genomic instability associated with *SMARCA4* deficiency.

A distinctive aspect of this case was the initial presentation with SVC syndrome (SVCS). At diagnosis (May 2023), symptoms included exertional dyspnea, facial edema, and prominent chest wall veins. Imaging confirmed a central right hilar mass with suspected vascular invasion (right brachiocephalic and azygos veins), directly causing SVCS. Initial radiotherapy (50 Gy/25 fractions) led to rapid tumor shrinkage and significant symptomatic relief, providing a critical window for subsequent systemic therapy.

Systemic treatment strategies for *SMARCA4*-dNSCLC remain under investigation, with current evidence largely derived from retrospective studies and case reports. Literature consistently indicates that NSCLC harboring *SMARCA4* mutations or SWI/SNF complex deficiency is typically associated with a high tumor mutation burden (TMB), a key biomarker for responsiveness to immune checkpoint inhibitors (ICIs) (1, 9, 15). Specifically, Schoenfeld et al. noted that while these tumors often exhibit lower or negative PD-L1 expression, they are characterized by elevated TMB. Emerging research suggests that this high TMB (≥10 mut/Mb) correlates with improved progression-free survival and overall survival in patients receiving immunotherapy. Consequently, the elevated TMB profile provides a mechanistic rationale for the potential efficacy of ICIs in this subgroup. However, the predictive value of TMB in this population is nuanced. While high TMB suggests immunogenicity, its practical utility can be compromised by co-occurring mutations in *STK11* and *KEAP1*, which are frequent in SMARCA4-dNSCLC and are known to drive a “cold” immune microenvironment and primary resistance to ICIs.

In the present case, PD-L1 expression and TMB status were not evaluated due to financial limitations. Nevertheless, we proceeded with an ICI-based regimen tailored to the molecular features of *SMARCA4*-dNSCLC. This therapeutic decision based on two key factors: first, *SMARCA4*-deficient tumors generally exhibit a high TMB, which serves as a surrogate marker for immunogenicity; and second, accumulating data suggest that ICIs (e.g., nivolumab or pembrolizumab) can confer significant survival benefits in this specific patient population, even in the absence of biomarker confirmation (2, 16–21).

Given the limitations of monotherapy, combination strategies integrating immunotherapy with chemotherapy have demonstrated promising therapeutic potential (2). While regimens incorporating pemetrexed have shown efficacy in individual cases (22, 23), retrospective analyses suggest that taxane-based chemoimmunotherapy regimens are associated with longer progression-free survival (PFS) compared to pemetrexed-based regimens (10.0 months vs. 7.3 months, P < 0.001) (8). This evidence supported our selection of a nab-paclitaxel-based regimen for this patient.

Regarding therapeutic alternatives, the role of anti-angiogenic agents in *SMARCA4*-dNSCLC remains an area of exploration. While regimens incorporating bevacizumab (such as IMpower150) have demonstrated efficacy in certain *EGFR*-mutated or metastatic NSCLC cohorts (23). direct evidence for their use in the specific setting of acquired *EGFR* mutations following multiline therapy is currently limited. In the present case, despite the theoretical potential of anti-angiogenic strategies, these agents were strictly withheld following a careful risk-benefit evaluation. This decision was primarily driven by the high risk of fatal hemorrhage associated with the tumor’s direct invasion into the superior vena cava and the recurrent SVCS. Consequently, the patient did not receive anti-angiogenic therapy.

Building on these biological complexities, biomarker selection remains a significant challenge. A 2023 retrospective analysis (24) highlighted that although these tumors generally exhibit high TMB, they often demonstrate low PD-L1 expression and a high frequency of HLA-I heterozygosity loss. Consequently, some patients show poor responses even to combination immunotherapy. This confirms that TMB and PD-L1 may not be ideal standalone predictive biomarkers for efficacy in SMARCA4-deficient tumors due to the aforementioned genomic interference from co-mutations, underscoring the urgency of identifying more reliable markers to optimize treatment strategies.

Targeted therapy options for *SMARCA4*-deficient tumors are generally limited, as these malignancies typically harbor a smoking-associated mutational signature (e.g., *TP53*, *STK11*, *KEAP1*) and lack actionable driver mutations. Large-scale genomic profiling indicates that *SMARCA4* alterations and *EGFR* mutations are exceedingly rare co-occurrences (1, 8); for instance, among 59 cases of *SMARCA4*-deficient tumors, only one exhibited an *EGF*R L858R mutation.

However, the present case vividly illustrates the phenomenon of therapy-induced clonal evolution. Although the initial NGS was negative, we hypothesize that the *EGFR L858R* mutation likely existed as a minor subclone below the assay’s limit of detection (LOD) at diagnosis, rather than arising *de novo*. It is plausible that the initial multimodal treatment successfully suppressed the dominant EGFR-wildtype clones, creating a selective pressure that allowed this pre-existing, undetectable minor subclone to expand and become the dominant driver.

Upon detection of this mutation via NGS, Aumolertinib was selected as the therapeutic agent. The rationale for this choice was twofold: first, Aumolertinib is a standard-of-care third-generation EGFR-TKI in China with a safety and efficacy profile comparable to Osimertinib; second, its superior potency against L858R mutations and high blood-brain barrier penetration were deemed critical given the patient’s advanced stage and risk of metastasis.

This strategy mirrors a recent report where a patient with *SMARCA4* loss and concurrent EML4-ALK fusion achieved a sustained response to ALK-TKI therapy (25). Collectively, these findings indicate that despite the rarity of targetable drivers, dynamic and comprehensive genomic profiling remains critical for identifying actionable alterations (e.g., in *ALK* or *EGFR*) and guiding precision oncology in this challenging disease.

The value of radiotherapy for local control deserves reconsideration in this disease. Given that *SMARCA4*-deficient tumors are often centrally located and prone to forming bulky masses, patients frequently present with oncologic emergencies, such as superior vena cava syndrome (SVCS), at initial diagnosis. Considering the generally poor response to chemotherapy and the current scarcity of high-level evidence regarding radiotherapy, the experience from this case is particularly valuable. Among all modalities employed, radiotherapy induced the most significant tumor regression. It not only rapidly alleviated local symptoms and managed critical complications like SVCS but also secured a crucial therapeutic window for subsequent interventions, especially in patients with high tumor burden. This confirms that radiotherapy can serve as an irreplaceable and effective modality for managing oncologic emergencies or achieving local control in specific clinical scenarios.

This case, together with the existing literature, highlights a dual challenge in the management of *SMARCA4*-dNSCLC. On one hand, immunotherapy—particularly in combination with chemotherapy—demonstrates potential, though its efficacy is often variable, underscoring the need for careful patient selection. On the other hand, although targeted therapy is typically not applicable due to the mutual exclusivity with driver mutations, rare driver alterations (such as *EGFR*) may become detectable following multi-line therapy due to clonal evolution. This phenomenon offers salvage therapeutic opportunities for a subset of patients. Given the limited therapeutic arsenal, dynamic molecular profiling is of paramount importance to identify such emergent targets in the clinical management of this disease.

Regarding risk assessment in treatment decisions, anti-angiogenic agents were withheld in this case following a careful risk-benefit evaluation due to the potential hemorrhage risk associated with recurrent SVCS. Although subsequent options such as etoposide or antibody-drug conjugates (ADCs) remained theoretically viable alternatives, the patient unfortunately did not receive further systemic therapy and ultimately succumbed to disease progression.

Substantial unknowns remain in the understanding and treatment of *SMARCA4*-dNSCLC. Clinicians should not be constrained by conclusions drawn from previous small-scale studies but should instead adopt a proactive approach, integrating all accessible treatment modalities in a patient-tailored manner. This case illustrates that early radiotherapy, as an individualized intervention, helped extend survival, suggesting that personalized strategies may at times exceed the scope of conventional guidelines in rare tumors such as this.

Finally, we sincerely express our gratitude to the patient and the family for their consistent trust and support throughout the treatment course.

## Data Availability

The raw data supporting the conclusions of this article will be made available by the authors, without undue reservation.
